# Partially Disordered Crystal Phases and Glassy Smectic Phases in Liquid Crystal Mixtures

**DOI:** 10.3390/ma18133085

**Published:** 2025-06-29

**Authors:** Aleksandra Deptuch, Anna Drzewicz, Magdalena Urbańska, Ewa Juszyńska-Gałązka

**Affiliations:** 1 Institute of Nuclear Physics, Polish Academy of Sciences, Radzikowskiego 152, PL-31342 Kraków, Poland; anna.drzewicz@ifj.edu.pl (A.D.); ewa.juszynska-galazka@ifj.edu.pl (E.J.-G.); 2 Institute of Chemistry, Military University of Technology, Kaliskiego 2, PL-00908 Warsaw, Poland; magdalena.urbanska@wat.edu.pl; 3 Research Center for Thermal and Entropic Science, Graduate School of Science, Osaka University, Osaka 560-0043, Japan

**Keywords:** plastic crystals, liquid crystals, smectic phases, glass transition, alpha relaxation

## Abstract

Three liquid crystalline mixtures were investigated, consisting of compounds abbreviated as MHPOBC and 3F5FPhF6 with molar ratios 0.9:0.1 (MIX5FF6-1), 0.75:0.25 (MIX5FF6-2), and 0.5:0.5 (MIX5FF6-3). The presence of the smectic A*, smectic C*, and smectic C_A_* phases was observed in all mixtures. The hexatic smectic X_A_* phase, present in pure MHPOBC, disappeared quickly with an increasing admixture of 3F5FPhF6. Vitrification of smectic C_A_* was observed for the equimolar mixture, with the glass transition temperature and fragility index comparable to the pure glassforming 3F5FPhF6 component. Partial crystallization to conformationally or orientationally disordered crystal phases was observed on cooling in two mixtures with a smaller fraction of 3F5FPhF6. Broadband dielectric spectroscopy was applied to study the relaxation times in smectic and crystal phases. Vogel–Fulcher–Tammann, Mauro–Yue–Ellison–Gupta–Allan, and critical-like models were applied for analysis of the α-relaxation time in supercooled smectic X_A_* and smectic C_A_* phases.

## 1. Introduction

**Figure 1:**
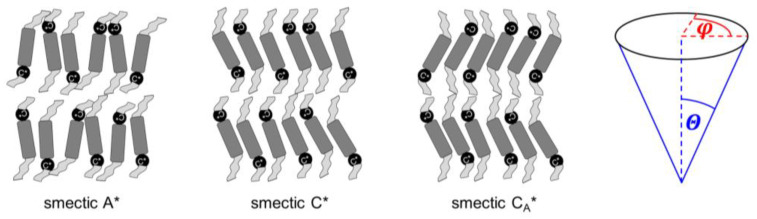
Schemes of the smectic A*, smectic C*, smectic C_A_* phases and definitions of the magnitude Θ and azimuth φ describing the tilt angle of molecules. The smectic X_A_* phase has a structure similar to that of smectic C_A_*, only with a hexatic bond-orientational order within the smectic layers. The tilt azimuth in smectic C*, C_A_*, X_A_* changes helically from layer to layer, with the helix pitch equal to hundreds of smectic layers.

Mesophases that appear for certain materials in the temperature range between the crystal and isotropic liquid phases include the conformationally-disordered crystals, plastic crystals with long-range positional order and some orientational disorder, and liquid crystals with long-range orientational order and some positional disorder [1,2]. The well-known types of liquid crystals include several smectic phases, which are all characterized by lamellar one-dimensional positional ordering and various two-dimensional ordering within the smectic layers [2]. In the simplest smectic phases A and C (SmA and SmC), the layer order is quasi-long-range, decay of which is described by the algebraic function [3], and the intra-layer order is short-range, where the decay is described by the exponential function [2]. The tilt angle of molecules in the smectic layers is described by the magnitude Θ and the azimuth (phase) φ [4] (Figure 1). The azimuth φ is random in the SmA phase and, consequently, the average tilt angle equals zero. However, the magnitude Θ can be larger than zero in the SmA phase. In such a case, the SmA → SmC transition occurs with a small layer shrinkage of ~1%; this type of SmA is named the de Vries SmA phase [5,6,7]. In the SmC phase, molecules in all smectic layers are on average tilted in the same direction, and the tilt angle differs from zero [5].

The asterisk * in the SmA* and SmC* notations means that phases are formed by chiral molecules (Figure 1). The SmC* phase has lower symmetry than the SmC phase formed by achiral molecules, which enables non-zero spontaneous polarization in a direction perpendicular to the tilt plane. Therefore, the SmC* phase can show bistable switching in an electric field, which was presented for the first time in the DOBAMBC compound (p-decyloxybenzylidene p’-amino 2-methyl butyl cinnamate) by Meyer et al. in 1975 [8]. The azimuth of the tilt changes by ca. 180° in the neighboring layers in the SmC_A_* phase, which shows tristable switching, presented for the first time in the MHPOBC compound by Chandani et al. in 1989 [9]. The synclinic order of the tilt angle in SmC* and anticlinic order in SmC_A_* are local, because at the longer scale, the tilt in both phases changes helically; the helix axis is perpendicular to the smectic layers and its pitch is usually 0.1–10 μm [5,10,11]. Thus, to obtain ferro- or antiferroelectric properties, it is necessary to use surface-stabilized samples, where the helix is unwound [5]. Other variants of tilted chiral smectic phases are SmC_α_* with a short helix pitch corresponding approximately to a few smectic layers, SmC_F1_*, where the azimuth of the tilt changes with a period of three smectic layers, and SmC_F2_*, where the azimuth of the tilt changes with a period of four smectic layers [4].

The bistable or tristable switching observed in the SmC* and SmC_A_* phases enables their application in liquid crystal displays [5,12,13]. The mesoscopic helical ordering in both phases provides selective reflection of light, which opens the possibility for application in thermography and optical filters [14,15,16]. The properties of liquid crystals, like the presence of particular phases, their temperature ranges, glassforming properties, electro-optic response, helix pitch, can be tuned in two ways, by modifications in the molecular structure [11,17,18] or by formulation of mixtures [11,19,20,21,22]. In this study, we focus on two liquid crystalline compounds that show different smectic phases on supercooling, which may consequently lead to the vitrification of different smectic phases in their mixtures, depending on the molar ratio of their components.

**Figure 2:**
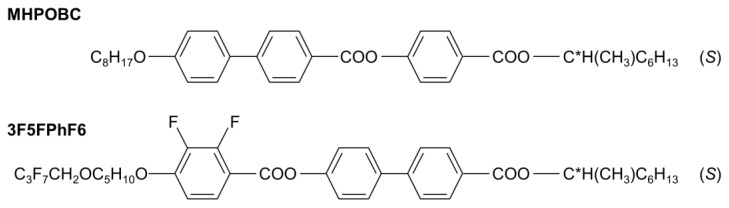
Molecular structures of the MHPOBC and 3F5FPhF6 compounds.

The subjects of this study were mixtures of (*S*)-4-[(1-methylheptyloxy)carbonyl]phenyl 4′-octyloxy-4-biphenylcarboxylate, abbreviated as MHPOBC, and (*S*)-4′-(1-methylheptyloxycarbonyl)biphenyl-4-yl 4-[5-(2,2,3,3,4,4,4-heptafluorobutoxy)pentyl-1-oxy]-2,3-difluorobenzoate, abbreviated as 3F5FPhF6 (Figure 2). The molecules of both compounds have the same structure of chiral terminal chain. The molecular cores consist of the three aromatic rings; however, the order of the phenyl and biphenyl parts in 3F5FPhF6 is reversed compared with MHPOBC. The length of the achiral terminal chain in 3F5FPhF6 is longer than in MHPOBC. Additionally, the 3F5FPhF6 molecules are fluorinated at the achiral terminal chain and in the phenyl ring. The phase sequences of these compounds on cooling are as follows:

MHPOBC: Iso (422 K), SmA* (395 K), SmC_α_* (394 K), SmC* (392 K), SmC_FI1_* (391 K), SmC_A_* (338 K), SmX_A_* [23,24,25];

3F5FPhF6: Iso (383 K), SmA* (381.5 K), SmC_α_* (381 K), SmC* (379.5 K), SmC_A_* (238 K), glSmC_A_* [26,27].

SmX_A_* refers to the hexatic smectic phase, which is characterized by the bond-orientational order; all domains with a short-range hexagonal order are oriented in the same direction in the smectic layer [2]. The melting temperature of MHPOBC is 357 K. Thus, the hexatic SmX_A_* phase is metastable, and the crystal phase eventually forms [25]. The melting temperature of 3F5FPhF6 is 326 K, and glass transition of the SmC_A_* phase is observed for cooling rates ≥ 10 K/min, while total or partial crystallization occurs on slower cooling [26,27].

This study aims to answer the following questions: (1) which fraction of 3F5FPhF6 leads to the disappearance of the hexatic SmX_A_* phase in a mixture? (2) which fraction of 3F5FPhF6 is necessary to obtain a mixture with good glassforming properties? (3) is it possible to obtain an MHPOBC/3F5FPhF5 mixture which has both glassforming properties and exhibits the SmX_A_* phase, which would enable investigation of relaxation processes in supercooled SmX_A_*? This study focused on an equimolar MHPOBC/3F5FPhF6 mixture, where glass transition of the smectic phase was expected, but mixtures with smaller molar fractions of 3F5FPhF6 (10% and 25%) were also tested. In the first step, the phase sequence of three mixtures was investigated by differential scanning calorimetry (DSC) for cooling and heating rates in the 2–40 K/min range. The interpretation of DSC thermograms was supported by polarizing optical microscopy (POM) observations performed at 10 K/min. In the second part of this study, the melt crystallization kinetics at various cooling rates were analyzed using the Augis–Bennett [28] and isoconversional methods [29,30,31]. In the third part, the structures of smectic phases were investigated by X-ray diffraction (XRD), including their layer spacing, average intermolecular distances, correlation length of the short-range order, and electron density profile in a direction perpendicular to the smectic layers. Finally, the relaxation processes in the smectic and crystal phases were studied via broadband dielectric spectroscopy (BDS), with a focus on the α-relaxation related directly to the glass transition [32,33,34,35]. The α-relaxation time was analyzed using the Vogel–Fulcher–Tammann (VFT) [32,34] and Mauro–Yue–Ellison–Gupta–Allan (MYEGA) [33] formulas; the glass transition temperature and the fragility index obtained via extrapolation of the α-relaxation time to 100 s by the VFT formula were compared with those obtained via the MYEGA formula. The applicability of the critical-like formula [35] was also tested.

## 2. Materials and Methods

The components of the mixtures, (*S*)-4-[(1-methylheptyloxy)carbonyl]phenyl 4′-octyloxy-4-biphenylcarboxylate (MHPOBC) [24] and (*S*)-4′-(1-methylheptyloxycarbonyl)biphenyl-4-yl 4-[5-(2,2,3,3,4,4,4-heptafluorobutoxy)pentyl-1-oxy]-2,3-difluorobenzoate (3F5FPhF6) [11], were synthesized in the Institute of Chemistry of the Military University of Technology in Warsaw. The synthetic route of MHPOBC is described in [36] and that of 3F5FPhF6 is described in [37]. Three mixtures were prepared, with the abbreviations and molar ratios as follows:

- MIX5FF6-1, MHPOBC:3F5FPhF6 molar ratio 0.8992(4):0.1008(3);
- MIX5FF6-2, MHPOBC:3F5FPhF6 molar ratio 0.7505(4):0.2495(3);
- MIX5FF6-3, MHPOBC:3F5FPhF6 molar ratio 0.4986(5):0.5014(4).

The weighted components were dissolved in acetone, and the solutions were mixed and kept on a hot plate set at ca. 313 K until the acetone evaporated. Then, each mixture was heated to 433 K to obtain the isotropic liquid and cooled back down to room temperature.

The TA Instruments DSC 2500 calorimeter (New Castle, DE, USA) was used for DSC measurements. The samples weighing 4.84, 9.51, and 6.47 mg for MIX5FF6-1, MIX5FF6-2, and MIX5FF6-3 were placed within aluminum pans. The DSC scans were carried out with cooling/heating rates of 2, 5, 8, 10, 15, 20, 25, 30, 35, 40 K/min in the cooling and subsequent heating cycle, maintaining the same rate between 173 K and 433 K. The thermograms were analyzed in the TRIOS program.

A Leica DM2700 P microscope (Wetzlar, Germany) was used for POM observations. The samples had a form of film between two thin glass slides without aligning layers. The POM textures were registered in 5 s intervals during cooling and heating at a 10 K/min rate in the 188–433 K range. Each image was subjected to numerical analysis. The TOApy program [38] was used to calculate the weighted mean intensity, and the ImageJ 1.52a program [39] calculated the mean red, green, and blue components.

A PANalytical X’Pert PRO diffractometer (Malvern, UK) incorporating an X-ray tube with a Cu anode ($\lambda_{CuK\alpha 1}$ = 1.540562 Å, $\lambda_{CuK\alpha 2}$ = 1.544390 Å [40]) was used for XRD measurements. The samples were placed in a 13 mm × 10 mm × 0.2 mm flat sample holder, and the XRD patterns were collected via Bragg–Brentano geometry. The measurements were performed during cooling in the 298–433 K range. The 2θ angle was calibrated using the NIST Standard Reference Material 675 [41], supplied by Merck (Darmstadt, Germany). Additionally, the remaining small systematic error in the peak positions was compensated based on the (002), (003), and (004) peak positions deep in the SmC_A_* phase. The WinPLOTR [42] and OriginPro 2020b programs were used for the XRD data analysis.

A Novocontrol impedance spectrometer (Frankfurt, Germany) was used to collect the dielectric spectra. Samples with a thickness of 50 μm were placed between gold electrodes with polytetrafluoroethylene spacers. The BDS measurements were performed in the 0.1–10^7^ Hz range in two temperature programs, as follows: (1) the sample was heated to 433 K and dielectric spectra were collected on slow cooling to 173 K and slow heating to 433 K; or (2) the sample was heated to 433 K, cooled fast to 173 K at 10 K/min, and dielectric spectra were collected on slow heating to 433 K. The BDS data analysis was conducted using the OriginPro 2020b program.

## 3. Results and Discussion

### 3.1. Phase Sequence

**Figure 3:**
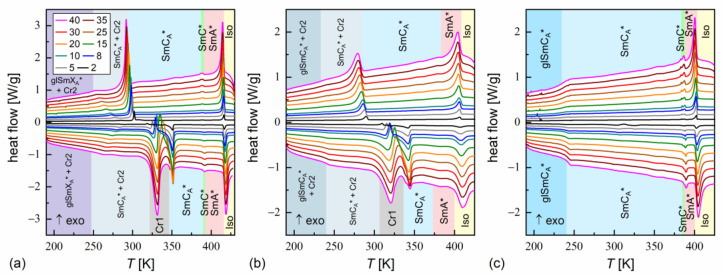
DSC thermograms of MIX5FF6-1 (**a**), MIX5FF6-2 (**b**), and MIX5FF6-3 (**c**) for various cooling/heating rates in K/min. The legend in (**a**) is common for all panels.

**Figure 4:**
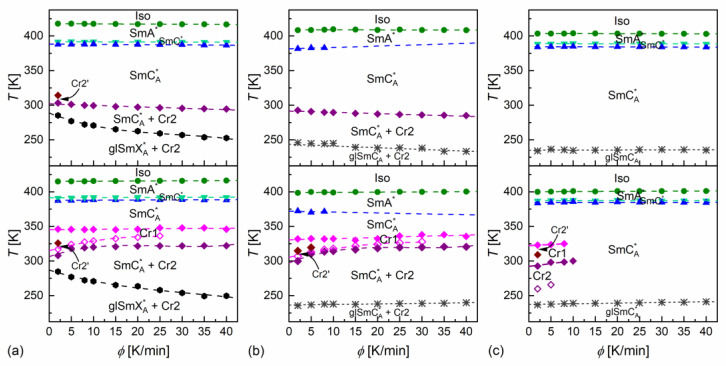
Phase transition temperatures of MIX5FF6-1 (**a**), MIX5FF6-2 (**b**), and MIX5FF6-3 (**c**) determined from the DSC thermograms for various rates of cooling (upper row) and heating (bottom row).

The phase transition temperatures were determined as the onset temperatures according to DSC thermograms [43]. An exception was the transition between the SmA* and SmC* phases, where the peak temperature was used in some cases, because the onset temperature was not obtained due to overlapped anomalies. The glass transition was determined as according to the middle height of the step in heat capacity [44]. The DSC results are presented in Figure 3 and Figure 4.

DSC thermograms revealed three anomalies above the melting temperature for MIX5FF6-1 and MIX5FF6-3, which were attributed to the Iso/SmA*/SmC*/SmC_A_* transitions. The transition temperatures for MIX5FF6-1, extrapolated to 0 K/min, were Iso (417.6 K), SmA* (391.6 K), SmC* (388.1 K), SmC_A_* on cooling and SmC_A_* (387.6 K), SmC* (391.3 K), SmA* (414.9 K), Iso on heating. The enthalpy change at the Iso/SmA* transition was 6.0 kJ/mol, and the summed enthalpy change at the SmA*/SmC*/SmC_A_* transitions was 0.3 kJ/mol. The transition temperatures for MIX5FF6-3 were Iso (403.5 K), SmA* (388.8 K), SmC* (384.5 K), SmC_A_* on cooling and SmC_A_* (384.5 K), SmC* (387.3 K), SmA* (400.0 K), Iso on heating. The enthalpy changes at the Iso/SmA*, SmA*/SmC*, and SmC*/SmC_A_* transitions were 5.7 kJ/mol, 0.6 kJ/mol, and 0.2 kJ/mol, respectively. For the MIX5FF6-2 mixture, the anomaly between the SmA* and SmC_A_* phases was broad, and the onset temperatures were determined only for 2–10 K/min rates. The phase sequence was Iso (408.8 K), SmA* (381.4 K), SmC_A_* on cooling and SmC_A_* (371.9 K), SmA* (398.9 K), Iso on heating. The enthalpy changes at the Iso/SmA* and SmA*/SmC_A_* transitions were 5.4 kJ/mol and 0.3 kJ/mol, respectively. SmA*/SmC* transition was not observed. However, the DSC results for other mixtures suggest that the SmC* phase should also have been present in MIX5FF6-2. The SmC_α_* phase, reported for pure components in very narrow temperature ranges [24,26], was not observed in MIX5FF6-1, -2, -3.

The DSC results indicated increasing glassforming properties with the increasing fraction of the 3F5FPhF6 component. MIX5FF6-1 crystallized at 294–301 K during cooling at 5–40 K/min and formed a crystal phase denoted as Cr2. With the lowest 2 K/min cooling rate, the crystallization started at 314 K, and a mix of Cr2 and additional Cr2’ phase was formed. On further cooling, there was an anomaly in the form of a peak for lower cooling rates that gradually transformed into a step-like anomaly as the rate of cooling increased. This was interpreted as the transition from the remaining SmC_A_* phase to the more ordered, hexatic SmX_A_* phase. Consequently, the step in heat capacity observed for higher cooling rates represented the glass transition of the SmX_A_* phase. The Cr2 phase melted at 308–322 K and was followed by cold crystallization to another crystal phase, denoted as Cr1, which melted at 346–348 K. With the 2 K/min heating rate, there was an additional endothermic anomaly at 326 K, attributed to the melting of the Cr2’ phase. In the POM observations carried out at 10 K/min, the MIX5FF6-1 sample showed mainly homeotropic alignment (with the smectic layers parallel to the sample’s surface) with only small areas of fan-shaped texture (Figures S1 and S2 in Supplementary Materials). Because of that, the transitions between the smectic phases were not reflected in the results of the numerical analysis, but visual inspection of the textures enabled distinction between the SmA*, SmC*, and SmC_A_* phases. In contrast, the helix inversion at 347 K on cooling led to a peak in the luminance. The crystallization that occurred, probably parallel to the SmC_A_* → SmX_A_* transition, revealed a step in the blue textural component. The glass transition of SmX_A_* did not lead to any abrupt change in the texture, but at low temperatures, fractures in the form of dark lines on the homeotropically aligned area were observed (see textures collected at 200 K in Figure S1 and at 230 K in Figure S2 in the Supplementary Materials). Such fractures are sometimes observed in the glassy state [45,46]. A small step in the luminance at ~328 K, observed on heating, was attributed to the melting of Cr2 and the following cold crystallization of Cr1. A more significant increase in luminance at 351 K corresponded to melting of the Cr1 phase.

MIX5FF6-2 crystallized at 285–293 K to the Cr2 phase. At 233–246 K, the remaining SmC_A_* phase underwent glass transition. There was no anomaly indicating the SmC_A_*/SmX_A_* transition. Glass softening during heating occurred at 236–240 K. The Cr2 phase melted at 310–320 K, followed by cold crystallization to Cr1, melting at 332–338 K. At the 2 and 5 K/min heating rates, there were additional melting anomalies at 315 K and 320 K, respectively, corresponding to the melting of the Cr2’ phase. The POM textures of MIX5FF6-2 were fan-shaped (Figures S3 and S4 in the Supplementary Materials). The phase transitions between the smectic phases and the crystallization and melting of the Cr2 and Cr1 phases were visible as changes in color. Most importantly, the SmC* phase, which DSC did not detect, was observed via POM between SmA* and SmC_A_*, both on cooling and heating. The glass transition of SmC_A_* did not lead to changes in the POM textures. In both MIX5FF6-1 and MIX5FF6-2, the crystallization did not distort the fan-shaped texture typical of smectic phases [47], and growing crystallites were not directly observed, indicating that the sizes of these were very small.

MIX5FF6-3 did not crystallize at cooling rates of 2–40 K/min. The glass transition of the SmC_A_* phase occurred at 234–237 K during cooling and at 237–241 K during heating. Exothermic anomalies related to cold crystallization were visible for the 2 and 5 K/min heating rates. However, cold crystallization also occurred at the 8 and 10 K/min heating rates, and melting anomalies were observed during further heating. The Cr2 and Cr1 phases melted at 293–300 K and 323–325 K, respectively. The Cr1 phase did not form at 10 K/min. At 2 K/min, an additional Cr2’ phase melted at 309 K. The melting anomalies of the main Cr2 and Cr1 phases in all mixtures resembled those observed for pure MHPOBC [48], with melting temperatures decreasing with the increasing fraction of 3F5FPhF6. The fan-shaped POM textures of MIX5FF6-3 confirmed the presence of the SmA*, SmC*, and SmC_A_* phases (Figures S5 and S6 in the Supplementary Materials). A broad minimum in the blue component within the SmC_A_* phase was centered at ~310 K on cooling and ~330 K on heating; this was probably related to the helix inversion. Similarly, as for MIX5FF6-2, the textures in the glassy state did not show any fractures; they were observed only in the homeotropic texture of MIX5FF6-1.

### 3.2. Crystallization Kinetics

**Figure 5:**
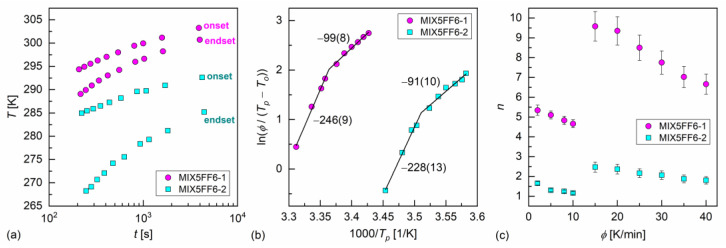
Continuous cooling–transition diagram (**a**), Augis–Bennett activation plot (**b**), and Avrami parameter (**c**) of MIX5FF6-1 and MIX5FF6-2. The effective activation energies in (**b**) are given in kJ/mol.

The melt crystallization of MIX5FF6-1 and MIX5FF6-2 at various cooling rates was investigated based on the DSC thermograms (Figure 3a,b). The continuous cooling–transition (CCT) diagrams [49] for both mixtures are presented in Figure 5a. The onset temperature of the crystallization of MIX5FF62 was ~10 K lower than for MIX5FF6-1 at the same cooling rate. The endset temperature [43] of the crystallization of MIX5FF6-2 shifted by ~15–20 K to lower temperatures compared with MIX5FF6-1. Thus, the crystallization of MIX5FF6-2 was spread over a wider temperature range. The Augis–Bennett model [28] and the isoconversional method [29,30,31] were applied to determine the effective activation energy of crystallization. The Augis–Bennett model focuses on the initial and intermediate parts of crystallization [28], while the isoconversional method provides effective activation energy for a selected degree of crystallization [29,30,31]. The activation plot in the Augis–Bennett model is based on the following formula [28]:

(1) $$ln\left(\frac{\phi }{T_{p}-T_{o}}\right)=C_{AB}-\frac{E_{AB}}{RT_{p}},$$

where ϕ is the rate of temperature change, $T_{p}$ and $T_{o}$ are peak and onset temperatures of the exothermic anomaly related to crystallization, $E_{AB}$ is the effective activation energy, R is the gas constant, and $C_{AB}$ is a fitting parameter. The activation plots of MIX5FF6-1 and MIX5FF6-2 are shown in Figure 5b. Although they are shifted along the inverted temperature scale, their slopes and the corresponding activation energies are equal within uncertainties. The $E_{AB}$ values with slow cooling (2–10 K/min) were −246(9) kJ/mol and −228(13) kJ/mol for MIX5FF6-1 and MIX5FF6-2, respectively. With fast cooling (15–40 K/min), the $E_{AB}$ values were −99(8) kJ/mol for MIX5FF6-1 and −91(10) kJ/mol for MIX5FF6-2. The negative $E_{AB}$ means that the crystallization kinetics were constrained mainly by the rate of nucleation, especially at slow cooling, where the crystallization started at a higher temperature, further from the optimal temperature range of nucleation [31,50,51]. The Augis–Bennett analysis also enabled estimation of the Avrami parameter n, which provides information about the shape of growing crystallites [52]. The formula for n is as follows [28]:

(2) $$n=\frac{2.5RT_{p}^{2}}{E_{AB}{∆T}_{FWHM}},$$

where ${∆T}_{FWHM}$ is the full width at half-height of the exothermic anomaly related to crystallization. The obtained n values, shown in Figure 5c, were much lower for MIX5FF6-2 than for MIX5FF6-1 due to wider exothermic anomaly and lower temperature region of melt crystallization. For both mixtures, n was larger with fast cooling due to the lower absolute values of activation energy. In MIX5FF6-2, n = 1.2–1.6 and n = 1.8–2.5 with slow and fast cooling corresponded to the growth of one-dimensional and two-dimensional crystals, respectively [52]. In MIX5FF6-1, n = 4.7–5.3 at slow cooling indicated growth of spherical or sheaf-like crystals [52]. The Avrami parameter n = 6.7–9.6 with fast cooling was unusually high. Generally, n up to only 6 is expected [52], but higher values have also been reported [53,54]. In Ref. [54], very high n was interpreted as the formation of sheaf-like, weakly developed crystallites.

The activation plot in the isoconversional method is based on the following formula [29,30,31]:

(3) $$\frac{dx(t)}{dt}=f(x)A\exp\left(-\frac{E_{eff}}{RT_{x}}\right),$$

where x(t) is th conversion degree, $T_{x}$ is the temperature where a selected x is reached, $E_{eff}$ is the effective activation energy, f(x) and A are fitting parameters. The conversion degree is calculated from the DSC thermogram as follows:

(4) $$x\left(T\right)=\frac{\int_{T_{start}}^{T}\Phi \left(T\right)dT}{\int_{T_{start}}^{T_{end}}\Phi \left(T\right)dT},$$

where $\Phi \left(T\right)$ is the heat flow. Then, the conversion rate is obtained as follows:

(5) $$\frac{dx(t)}{dt}=\frac{dx(T)}{dT}\phi .$$

**Figure 6:**
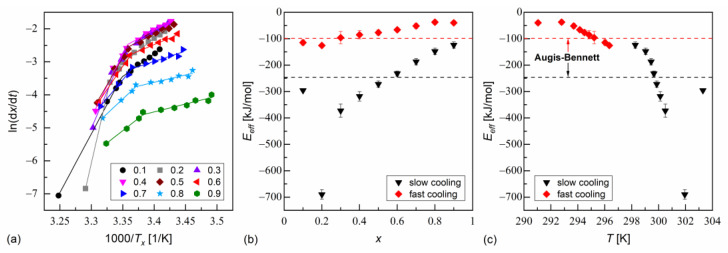
Isoconversional analysis of melt crystallization in MIX5FF6-1: activation plot (**a**), and effective activation energy vs. conversion degree (**b**) and average temperature (**c**).

**Figure 7:**
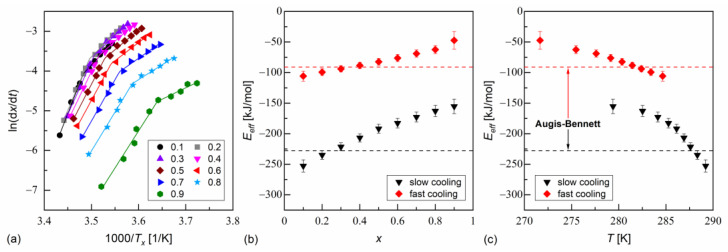
Isoconversional analysis of melt crystallization in MIX5FF6-2: activation plot (**a**), and effective activation energy vs. conversion degree (**b**) and average temperature (**c**).

The isoconversional analysis leads to conclusions consistent with the Augis–Bennett method (Figure 6 and Figure 7). The activation plots for conversion degrees from 0.1 to 0.9 show two linear regions, with a larger slope for slow cooling and a smaller slope for fast cooling. The effective activation energies were negative. Their absolute values decreased with increasing conversion degree and with the decreasing temperature. The thermodynamic driving force of crystallization increases with increasing supercooling [51]. Consequently, the nucleation constraints on the overall crystallization decrease. Only for MIX5FF6-1, in the initial stages of crystallization on slow cooling, $E_{eff}$ deviated from this trend; for x = 0.1 and 0.2, the activation energy was −296(5) kJ/mol and −690(18) kJ/mol, respectively. This was related to the crystallization of another Cr2’ phase, which formed on slow cooling next to Cr2 (Figure 4a).

### 3.3. Structure of Smectic Phases

**Figure 8:**
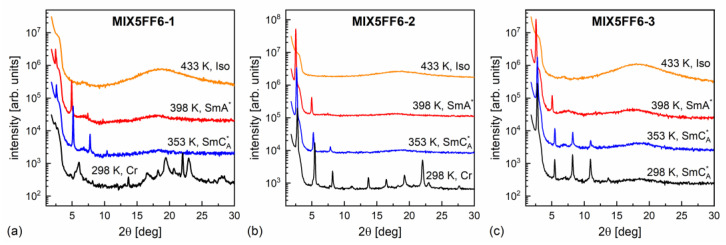
Representative XRD patterns of MIX5FF6-1 (**a**), MIX5FF6-2 (**b**), and MIX5FF6-3 (**c**) collected on cooling.

**Figure 9:**
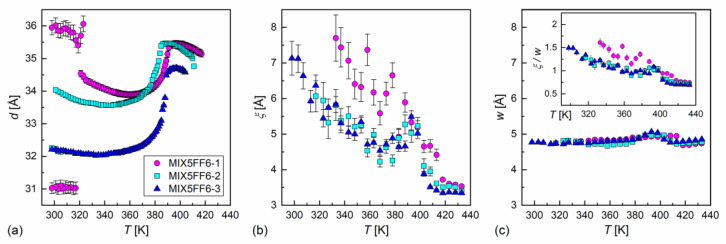
Smectic layer spacing (**a**), correlation length of the short-range order (**b**), and average inter-molecular distance (**c**) in the MIX5FF6-1, -2, -3 mixtures vs. temperature. For easier comparison of the correlation length and the intermolecular distance, the ratio of these variables is shown in the inset in (**c**); the same scale is used in the (**b**,**c**) panels.

The XRD patterns of MIX5FF6-1, -2, -3 mixtures in various phases are presented in Figure 8. The lamellar order in the smectic phases showed sharp peaks at low 2θ angles in the XRD patterns. Only the (001) and (002) peaks were observed in the SmA* phase, while additional (003) or even (003) and (004) peaks were visible deep in the SmC_A_* phase. The smectic layer spacing d is related to the (00l) peak position θ via the Bragg equation $l\lambda_{CuK\alpha 1}=2d\sin\theta$ [55]. The position of the (002) peak was used to determine the smectic layer spacing, because it was located on a much lower background than the (001) peak. For MIX5FF6-1, the (001) peak was used to determine the layer spacing below the onset of crystallization because of the low intensity of the (002) peak. The smectic layer spacing decreased with increasing amounts of the 3F5FPhF6 component (Figure 9a). The difference between MIX5FF6-1 and MIX5FF6-2 was small, while for MIX5FF6-3, a more significant decrease in the smectic layer spacing was observed. In contrast, the total layer shrinkage $(d_{A}-d_{C})/d_{C}$ increased with the increasing amount of 3F5FPhF6: 4.4(4)% for MIX5FF6-1, 5.4(1)% for MIX5FF6-2, and 7.7(1)% for MIX5FF6-3. The $d_{A}$ value is the maximal layer spacing in the SmA* phase and $d_{C}$ is the minimal layer spacing in the SmC_A_* phase. The 4–8% layer shrinkage was larger than ~1%, corresponding to the de Vries SmA* phase. However, a larger border value of 4–5% is sometimes mentioned [56,57]. If this broader range is considered, then the SmA* phase of MIX5FF6-1 was at the border between conventional and de Vries SmA*, while in other mixtures it was a conventional SmA* phase. A lower level of layer shrinkage reduces the number of defects upon transition to the tilted smectic phase [5]. In MIX5FF6-1, the smectic layer spacing increased during crystallization to values larger than in the SmA* phase. This indicates the transition from the remaining SmC_A_* fraction to the hexatic SmX_A_* phase [48], which was suggested by the DSC thermograms.

The short-range positional order in the isotropic liquid and inside the smectic layers was shown in the XRD patterns with a wide maximum at 2θ ≈ 18°. The shape of this maximum is described by the Lorentz peak function in the space of the scattering vector $q=4\pi \sin\theta /\lambda_{CuK\alpha 1}$ [58], as follows:

(6) $$I\left(q\right)=\frac{A}{1+\xi^{2}{\left(q-q_{0}\right)}^{2}}+Bq+C,$$

where $q_{0}$ is the maximum position, ξ is the correlation length of the short-range order, A is the height of the maximum, and B and C are the slope and intercept, respectively, of the linear background. The average intermolecular distance is obtained as $w=2\pi /q_{0}$. The correlation length in the isotropic liquid phase was ca. 3.5 Å in all the mixtures and increased below the Iso → SmA* transition (Figure 9b). The ξ values in the smectic phases were larger in MIX5FF6-1 than in MIX5FF6-2 and MIX5FF6-3 at the same temperatures, in agreement with the hexatic SmX_A_* phase arising at lower temperatures only in MIX5FF6-1. A local maximum in ξ at ca. 390 K and a local minimum at ca. 370 K were noted for MIX5FF6-2 and MIX5FF6-3, and the temperature dependences of ξ practically overlapped for these two mixtures until the crystallization of MIX5FF62. The maximal observed correlation lengths were 7.7(7) Å, 6.1(5), and 7.1(5) Å for MIX5FF6-1, -2, -3, respectively. The average intermolecular distance of 4.7–5.1 Å showed a weaker dependence on temperature than the correlation length (Figure 9c). The local maximum in w was observed in the SmA* phase for all mixtures. The ξ/w ratio equalled 0.7–1.5 (inset in Figure 9c), which was interpreted as indicating correlations only between the nearest-neighbor positions. Decreased correlation length in MIX5FF6-2, -3 compared was MIX5FF6-1 was probably caused by the larger fraction of 3F5FPhF6 fluorosubstituted at the benzene ring. Recent computational results [59] show that such fluorosubstitution leads to larger disorder in dipole moments, which may consequently lower the short-range order within layers.

The electron density distribution along the smectic layer normal was symmetric, as molecules rotated along their short axes [60]; it can be described by the following series of cosine functions [61]:

(7) $$\rho \left(z\right)=\rho_{0}+\sum_{l=1}^{4}\pm |F_{00l}|\cos\left(2\pi lz/d\right),$$

where $F_{00l}$ are structure factors, the absolute values of which are obtained from the integrated intensities of the (00l) diffraction peaks from the smectic layer order, $\left|F_{00l}\right|=\sqrt{I_{00l}/Lp}$ [55]. The Lorentz–polarization correction Lp has different forms depending on the experimental conditions. For the powder samples without the preferred orientation takes the following form [62]:

(8) $$Lp\propto \frac{1+{cos}^{2}(2\theta )}{{sin}^{2}\theta \cos\theta }.$$

In contrast, for single crystal samples, the formula for $L_{p}$ is as follows [55]:

(9) $$Lp\propto \frac{1+{cos}^{2}(2\theta )}{\sin\left(2\theta \right)}.$$

**Figure 10:**
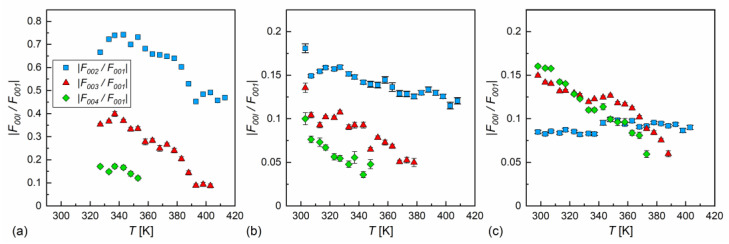
Absolute ratio of structural factors $F_{00l}$, where l = 2, 3, 4, and the main structural factor $F_{001}$, describing the electron density profile along the smectic layer normal in MIX5FF6-1 (**a**), MIX5FF6-2 (**b**), and MIX5FF6-3 (**c**). Note a much broader scale in (**a**) than in (**b**,**c**).

**Figure 11:**
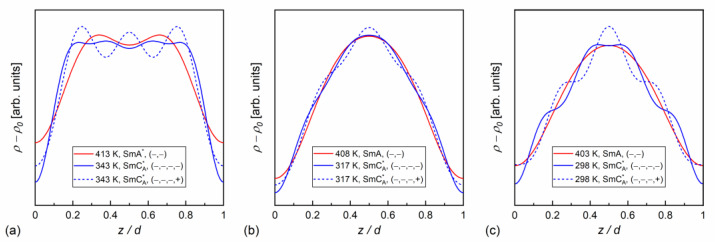
Electron density profiles along the smectic layer normal determined from the XRD patterns for MIX5FF6-1 (**a**), MIX5FF6-2 (**b**), and MIX5FF6-3 (**c**).

In our case, the samples were polycrystalline, but with a strong preferred orientation; on cooling from the isotropic liquid, the samples aligned homeotropically. This was recognized through the height of low-angle peaks, which were usually larger than the wide maximum at higher angles. In Ref. [60], Lp∝1/sinθ was applied for the homeotropically aligned smectic phase, which is the simplified form of Equation (9) for small θ. In our calculations, we applied Equation (9) in the full form. The absolute $F_{00l}$ values for l = 2, 3, 4, scaled by the $F_{001}$ factor, are presented in Figure 10. The $F_{001}$ factor was dominant in MIX5FF6-2 and MIX5FF6-3, as the $\left|F_{00l}/F_{001}\right|$ ratios were below 20%. This means that the $\rho \left(z\right)$ distribution can be described mainly by the cos(2πz/d) function, only weakly modified by higher harmonics. The situation was very different in MIX5FF6-1, where the $F_{002}$ and $F_{003}$ strongly contributed to the $\rho \left(z\right)$ distribution, with the $\left|F_{002}/F_{001}\right|$ and $\left|F_{003}/F_{001}\right|$ ratios deep in the SmC_A_* phase reaching almost 0.75 and 0.4, respectively. The XRD patterns did not provide information regarding the signs of the $F_{00l}$ factors and they were selected based on the assumption that the minimal $\rho \left(z\right)$ would be obtained at the border of the smectic layers (z = 0 and z = 1). Based on the $F_{001}$, $F_{002}$, and $F_{003}$ values for MIX5FF6-1, (−,−,−) signs were selected and applied to all mixtures to calculate $\rho \left(z\right)-\rho_{0}$, as presented in Figure 11. The sign for $F_{004}$ can be either (+) or (−). The results for MIX5FF6-3 in 298 K suggest that the (−,−,−,−) model suited the actual distribution well (Figure 11c). The increasing amount of the 3F5FPhF6 compound with fluorinated terminal chain (Figure 1) is expected to have contributed to the higher electron density close to the border of the smectic layer. This was reproduced better by the (−,−,−,−) model than the (−,−,−,+) one.

### 3.4. Dielectric Relaxation Processes

**Figure 12:**
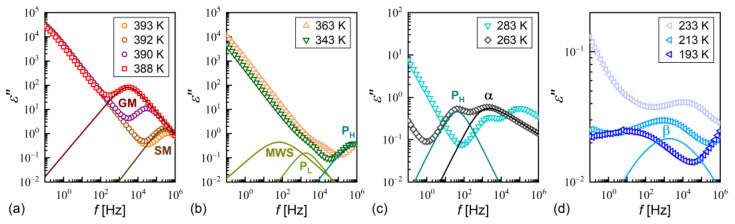
Dielectric absorption of MIX5FF6-3 vs. frequency measured on heating after fast cooling in the SmA* (390–393 K) and SmC* (388 K) phases (**a**), SmC_A_* phase (**b**,**c**), and SmC_A_* glass (**d**). The fitting results are presented separately for each process and with omitted low-frequency background, for 393 K and 388 K in (**a**), 343 K in (**b**), 263 K in (**c**), and 213 K in (**d**).

The dielectric spectra show the complex dielectric permittivity $\varepsilon^{*}$ as a function of frequency of the weak external electric field. Permittivity consists of the dispersion (real) part $\varepsilon^{\prime }$ and absorption (imaginary) part $\varepsilon^{\prime \prime }$: $\varepsilon^{*}\left(f\right)=\varepsilon^{\prime }\left(f\right)-i\varepsilon^{\prime \prime }\left(f\right)$, where i is the imaginary unit [63]. Representative dielectric spectra for different smectic phases and the SmC_A_* glass of MIX5FF6-3 are shown in Figure 12. The dielectric strength ∆ε and relaxation time τ of each process correspond to the area and position, respectively, of the peak in the dielectric absorption $\varepsilon^{\prime \prime }$ [63]. The relaxation processes observed in MIX5FF6-1, -2, -3 above the melting temperature were as follows:

- the soft mode, SM (Figure 12a), related to fluctuations in the magnitude Θ of the tilt angle (see Figure 1), with a small dielectric increment and relaxation time increasing with decreasing temperature in the SmA* phase [22,64,65];
- the Goldstone mode, GM (Figure 12a), related to fluctuations in the phase φ of the tilt angle, with a large dielectric increment and relaxation time weakly changing with temperature in the SmC* phase [22,64,65];
- P_L_ and P_H_ processes (Figure 12b), related to in-phase and anti-phase fluctuations in the phase φ of the tilt, typical for the SmC_A_* phase, with a much smaller dielectric increment than for GM and relaxation time increasing with decreasing temperature; the P_L_ process overlapped with rotations around the short molecular axis (s-process) [64,65,66].

An additional relaxation process was observed in some spectra at low frequencies in the smectic phases in the heating runs above the melting temperature; this may have been the Maxwell–Wagner–Sillars (MWS) process at the interface between the electrode and liquid crystal, which would fit well with the Cole–Cole model (a peak in ε” at the same frequency as a half-step in ε’) [67]. The MWS process was included in the fitting mainly as a background contribution to fit the nearby P_L_ process properly. Two relaxation processes were observed in the supercooled SmC_A_* phase in MIX5FF6-3, as follows:

- The primary α-process (Figure 12c) had a relaxation time that increased with decreasing temperature, usually in the super-Arrhenius manner, and reached 100 s at the glass transition temperature [32,34]. The α-relaxation has different origins in various substances [32,35,45,68,69] and in the SmC_A_* phase, it can be related to rotations around the molecular long axis. The dielectric strength of the α-process is comparable to that of the P_H_ process;
- The secondary β-process (Figure 12d) involved a smaller dielectric increment than the α-process and showed an Arrhenius dependence of the relaxation time on temperature, originating either from movements of the rigid molecules (genuine Johari–Goldstein relaxation) or from intramolecular rotation within flexible molecules (pseudo-Johari–Goldstein relaxation) [69].

**Figure 13:**
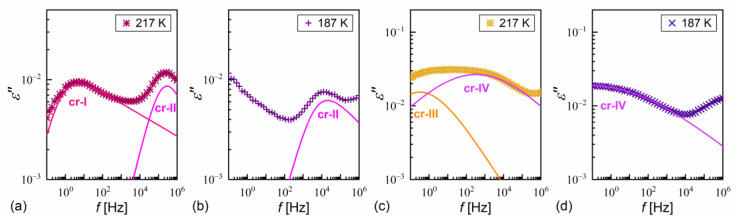
Dielectric absorption of MIX5FF6-1 vs. frequency measured on slow cooling (**a**,**b**) and heating after fast cooling (**c**,**d**) in the crystal phases.

α-relaxation was also observed in MIX5FF6-1 and MIX5FF6-2, while β-relaxation is not observed in these mixtures, probably due to the presence of stronger relaxation processes from the partially disordered crystal phases. The exemplary dielectric spectra in crystal phases are shown in Figure 13 for MIX5FF6-1; the same processes were present in the crystal phases of MIX5FF6-2. The processes denoted as crI (Figure 13a) and crII (Figure 13a,b) were observed in the slowly cooled samples, thus, they were assigned to the Cr2’ phase, was revealed in the DSC results only for the lowest 2 K/min cooling rate (Figure 4); Cr2’ would be expected to develop in a larger fraction on even slower, gradual cooling during BDS measurement. The processes denoted as crIII (Figure 13c) and crIV (Figure 13c,d) were observed for the sample cooled at 10 K/min, and these were assigned to the Cr2 phase, as indicated by the DSC thermograms (Figure 4).

The relaxation times of each process in the dielectric spectra of MIX5FF6-1, -2, -3 were determined by fitting the Havriliak–Negami [70] model of complex permittivity, as follows:

(10) $$\varepsilon^{*}\left(f\right)=\varepsilon_{\infty }+\sum_{j}\frac{{∆\varepsilon }_{j}}{{\left(1+{\left(2\pi if\tau_{HNj}\right)}^{1-a_{j}}\right)}^{b_{j}}}+\frac{S_{1}}{{\left(2\pi f\right)}^{n_{1}}}-\frac{iS_{2}}{{\left(2\pi f\right)}^{n_{2}}},$$

where $\varepsilon_{\infty }=\varepsilon^{’}(f\to \infty )$, ∆ε is dielectric strength, $\tau_{HN}$ is the Havriliak–Negami relaxation time, a and b are shape parameters. The latter two terms describe the low-frequency background originating from the ionic conductivity, polarization, and tails of the low-frequency relaxation processes [63]. In the Havriliak–Negami model, the shape parameters a, b are in the (0,1) range and the relaxation time τ, corresponding to the position of the peak in $\varepsilon^{\prime \prime }\left(f\right)$, relates to the Havriliak–Negami relaxation time $\tau_{HN}$ as follows [63]:

(11) $$\tau =\tau_{HN}{\left(\sin\left(\frac{\pi \left(1-a\right)}{2+2b}\right)\right)}^{-\frac{1}{1-a}}{\left(\sin\left(\frac{\pi \left(1-a\right)b}{2+2b}\right)\right)}^{\frac{1}{1-a}}.$$

If b = 1, the Havriliak–Negami model becomes the simpler Cole–Cole model [71]. For a = 0 and b = 1, the Debye model is obtained [63]. In both the Cole–Cole and Debye models, $\tau =\tau_{HN}$. The SM, GM, MWG, PL, PH, β, cr-III, and cr-IV processes are described by either the Cole–Cole or Debye models (symmetric $\varepsilon^{\prime \prime }(\log_{10}f)$ peak), while α, cr-I, and cr-II processes are described by the Havriliak–Negami model (asymmetric $\varepsilon^{\prime \prime }(\log_{10}f)$ peak). Exemplary fits of Equation (10), omitting the low-frequency background, are presented as lines in Figure 12 and Figure 13.

**Figure 14:**
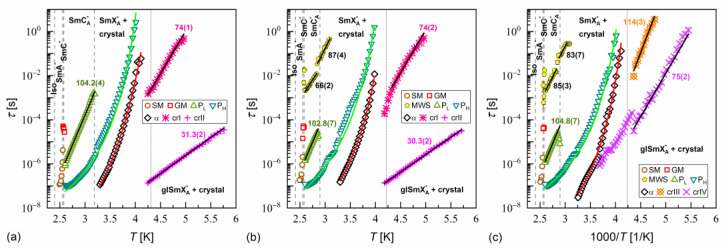
Relaxation times in MIX5FF6-1 determined from the BDS spectra collected on slow cooling (**a**), slow heating after slow cooling (**b**), and slow heating after fast cooling (**c**). The activation energies are given in kJ/mol.

**Figure 15:**
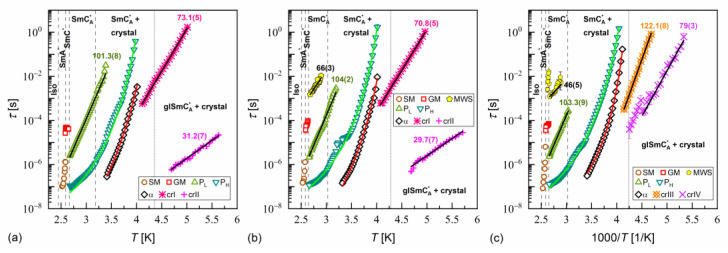
Relaxation times in MIX5FF6-2 determined from the BDS spectra collected on slow cooling (**a**), slow heating after slow cooling (**b**), and slow heating after fast cooling (**c**). The activation energies are given in kJ/mol.

**Figure 16:**
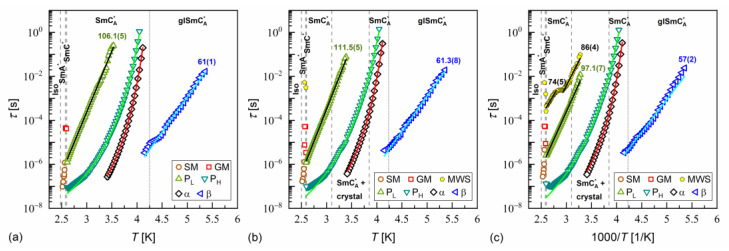
Relaxation times in MIX5FF6-3 determined from the BDS spectra collected on slow cooling (**a**), slow heating after slow cooling (**b**), and slow heating after fast cooling (**c**). The activation energies are given in kJ/mol.

The activation plots of relaxation times τ obtained from the BDS spectra for MIX5FF6-1, MIX5FF6-2, and MIX5FF6-3 are presented in Figure 14, Figure 15 and Figure 16, respectively. The relaxation times of P_L_, β, cr-I, cr-II, cr-III, and cr-IV processes showed linear dependence in the activation plots, thus, they followed the Arrhenius dependence on temperature, expressed as follows:

(12) $$\tau \left(T\right)=\tau_{\infty }\exp\left(\frac{E_{a}}{RT}\right),$$

where $\tau_{\infty }$ is the pre-exponential constant. The activation energy $E_{a}$ was equal to 97–112 kJ/mol for the P_L_ process, 57–61 kJ/mol for the β-relaxation, 71–74 kJ/mol for the cr-I process, 30–31 kJ/mol for the cr-II process, 114–122 kJ/mol for the cr-III process, and 75–79 kJ/mol for the cr-IV process. The MWS relaxation time showed Arrhenius behavior in some temperature ranges, with activation energy of 46–87 kJ/mol, varying between samples and temperature programs. The Arrhenius behavior and high $E_{a}$ of ca. 100 kJ/mol of the P_L_ process agree with its assumed overlap with the molecular s-process [22]. The even higher $E_{a}$ of the cr-III process indicates molecular reorientation around the short axis. The low $E_{a}$ of the cr-II process can be interpreted as indicative of intra-molecular conformational changes. Our earlier density functional theory calculations for a similar compound [72] imply that the most probable changes were in the C-O-C*-C or O=C-C-C torsional angles close to the chiral center (see Figure S3a,b in the Supplementary Materials of [72]). The β-relaxation was assigned to intra-molecular rotations of the benzene ring and biphenyl in the molecular core (see Figure S3b,c,e in the Supplementary Materials of [72]), the same as for pure 3F5FPhF6 [27]. The $E_{a}$ values of the cr-I and cr-IV processes were too high to assign them to any intra-molecular conformational changes [72], and a more probable origin of these processes is reorientation of molecules around their long axes. The conclusion is that the crystal phases observed in the BDS spectra of MIX5FF6-1 and MIX5FF6-2 were anisotropic plastic crystal phases.

The P_H_ and α-relaxation times showed a super-Arrhenius dependence on temperature, which can be described by the Vogel–Fulcher–Tamman (VFT) formula [32,34] as follows:

(13) $$\tau \left(T\right)=\tau_{\infty }\exp\left(\frac{B}{T-T_{V}}\right),$$

where $T_{V}$ is the Vogel temperature and τ→∞ and B correspond to the local activation energy divided by the gas constant. The α-relaxation time $\tau_{\alpha }$ is of particular interest, as it relates to the glass transition. The glass transition temperature $T_{g}$ corresponded to $\tau_{\alpha }$ = 100 s [32]. The deviation of $\tau_{\alpha }(T)$ from the Arrhenius dependence is related to the fragility index $m_{f}$ of the glassformer, as follows [32]:

(14) $$m_{f}={\left.\frac{d\log_{10}\tau_{\alpha }(T)}{d(T_{g}/T)}\right|}_{T=T_{g}}.$$

Glassformers with $m_{f}$ below and above 50 are assigned as strong and fragile, respectively [32,34]. If the $\tau_{\alpha }(T)$ values are extrapolated to low temperatures using the VFT formula, then $T_{g}$ and $m_{f}$ are determined as follows, with the assumption of SI units:

(15) $$T_{g}=T_{V}+\frac{B}{\ln100-\ln\tau_{\infty }},$$

(16) $$m_{f}=\frac{BT_{g}}{{\left(T_{g}-T_{V}\right)}^{2}ln10}.$$

The alternative formula for super-Arrhenius behavior is the Mauro–Yue–Ellison–Gupta–Allan (MYEGA) formula [33]:

(17) $$\tau \left(T\right)=\tau_{\infty }\exp\left(\frac{C}{T}\exp\left(\frac{K}{T}\right)\right),$$

where C is related to an effective activation barrier and number of topological degrees of freedom, and K is related to energy difference between constrained and unconstrained states. The advantage of the MYEGA model is that the relaxation time diverges at T = 0. In contrast, in the VFT model, $\tau_{\alpha }$ diverges at $T_{V}$ > 0, which is a matter of controversy [32]. If the $\tau_{\alpha }(T)$ values are extrapolated to low temperatures using the MYEGA formula, $T_{g}$ and $m_{f}$ can be expressed as follows:

(18) $$T_{g}=K{\left[W\left(\frac{K\left(\ln100-\ln\tau_{\infty }\right)}{C}\right)\right]}^{-1},$$

(19) $$m_{f}=\frac{C\left(1+\frac{K}{T_{g}}\right)\exp\left(\frac{K}{T_{g}}\right)}{T_{g}ln10},$$

where the Lambert W-function fulfills the inverse relationship $f\left(W\right)=W\exp W$ [73].

**Figure 17:**
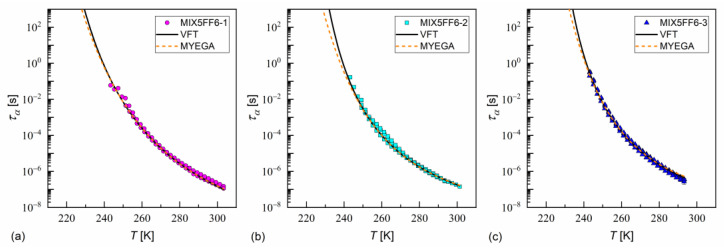
The α-relaxation time in MIX5FF6-1 (**a**), MIX5FF6-2 (**b**), and MIX5FF6-3 (**c**) as a function of temperature with the fitting results of the VFT and MYEGA formulas.

The fitting results of the VFT and MYEGA formulas to the α-relaxation times in MIX5FF6-1, -2, -3 are presented in Figure 17 and in Table 1 and Table 2, with the corresponding results for pure 3F5FPhF6 [27] for comparison. The $\tau_{\alpha }$ values for MIX5FF6-1 from Figure 14c were excluded from the analysis due to possible bias caused by overlap with the cr-IV process. Both the VFT and MYEGA models indicated that the glass transition temperature increased slightly with the increasing molar fraction of 3F5FPhF6, from 231–232 K for MIX5FF6-1 (10% 3F5FPhF6) to 238 K for pure 3F5FPhF6. The fragility index obtained by fitting the VFT model was 76 for MIX5FF6-1 and 105–109 for the other mixtures and 3F5FPhF6. The $m_{f}$ values obtained via the MYEGA model were lower and increased with the increasing molar fraction of 3F5FPhF6, from 67 for MIX5FF6-1 to 94 for pure 3F5FPhF6. Both models showed that MIX5FF6-1, -2, -3 and 3F5FPhF6 were fragile glassformers. According to Tanaka [74], stronger glassformers are characterized by the presence of a local order, which agrees with the lower $m_{f}$ of MIX5FF6-1, forming the glass of the hexatic SmX_A_* phase, and higher $m_{f}$ of MIX5FF6-2, MIX5FF6-3, and 3F5FPhF6, forming the glass of the less ordered SmC_A_* phase. On the other hand, Murthy [68] explained that an increase in $m_{f}$ resulted from an increasing number of hydrogen bonds between molecules. It should be noted that Ref. [74] considered metallic glassformers and Ref. [68] considered organic molecules containing an –OH group that is absent in MHPOBC and 3F5FPhF6 (although C-H…O bonds are possible [75]), and both references refer to the study of isotropic glasses. Thus, the relationship between $m_{f}$ and the 3F5FPhF6 fraction in these mixtures may have a different origin. Notably, the C parameter of the MYEGA model was one order of magnitude larger in the least fragile MIX5FF6-1 than in other samples, indicating either a larger effective activation barrier or a smaller number of topological degrees of freedom [33].

Another description of the α-relaxation time is the critical-like formula, expressed as follows [35]:

(20) $$\tau_{\alpha }\left(T\right)=A{\left(T-T^{*}\right)}^{-\gamma },$$

where A is a constant. The critical temperature $T^{*}$ and critical exponent γ are determined from the linear fit to the inverse of a derivative of $\ln\tau_{\alpha }(T)$:

(21) $${\left(\frac{d\ln\tau_{\alpha }(T)}{dT}\right)}^{-1}=\frac{T^{*}-T}{\gamma },$$

**Figure 18:**
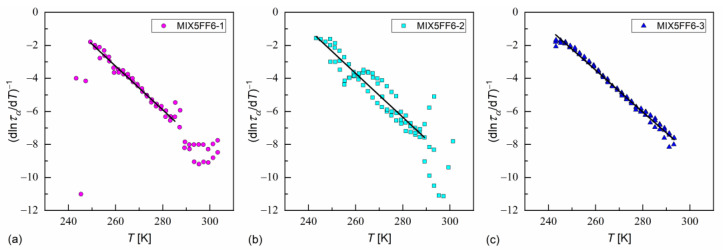
Determination of the critical temperature and critical exponent via linear fit to the inverse of a derivative of the logarithm of the α-relaxation time over temperature in MIX5FF6-1 (**a**), MIX5FF6-2 (**b**), and MIX5FF6-3 (**c**).

In Ref. [35], Starzonek et al. applied this analysis to glassformers with molecules possessing mainly uniaxial symmetry. This was only approximately fulfilled for MHPOBC and 3F5FPhF6 molecules, which were elongated, but which can take a nonlinear shape, e.g., hockey-stick [27,76,77]. Nevertheless, the length-to-width ratios obtained from the XRD results in the SmA* phase in MIX5FF6-1, -2, -3 were quite high (d/w ≈ 7); thus, Equation (21) describes well the $\tau_{\alpha }\left(T\right)$ dependence at low temperatures for MIX5FF6-1, MIX5FF6-2, and across the whole temperature range for MIX5FF6-3 (Figure 18, Table 3). The critical temperatures $T^{*}$ were close to the glass transition temperatures obtained from VFT and MYEGA models. The critical exponents were equal to γ = 7.5–7.9, slightly lower than 8.3–9.0 obtained in Ref. [35]. Lower γ ≈ 2 is expected at higher temperatures [35]; however, the data for MIX5FF6-1 and MIX5FF6-2 were too scattered to perform fitting above 285–289 K.

## 4. Conclusions

Liquid crystalline mixtures with different molar ratios of smectogenic MHPOBC and 3F5FPhF6 compounds were investigated, including MIX5FF6-1 (MHPOBC: 3F5FPhF6 0.9: 0.1), MIX5FF6-2 (0.75: 0.25), and MIX5FF6-3 (0.5: 0.5). The DSC, POM, XRD, and BDS results enabled us to answer the questions presented in the Introduction section. The conclusions are as follows:

(1) Based on the XRD results, the hexatic SmX_A_* phase was detected only in MIX5FF6-1, with a continuous increase in the layer spacing during the SmC_A_* → SmX_A_* transition and larger correlation length of the intra-layer short-range positional order in the preceding SmC_A_* phase compared with mixtures with a larger fraction of 3F5FPhF6;
(2) Only the equimolar MIX5FF6-3 mixture showed good glassforming properties in the smectic state; no crystallization was observed on cooling at 2–40 K/min. The crystal phase appeared only during heating in the process of cold crystallization. The glassforming properties of MIX5FF6-3 were better than pure 3F5FPhF6, where a cooling rate of at least 10 K/min was necessary to avoid partial crystallization [27]. MIX5FF6-1 and MIX5FF6-2, with smaller fractions of 3F5FPhF6, underwent partial melt crystallization on cooling and formed orientationally disordered (ODIC) crystal phases. However, the remaining fraction of SmX_A_* in MIX5FF6-1 and SmC_A_* in MIX5FF6-2 underwent glass transition. The glass transition temperatures were similar for all mixtures, 231–236 K, slightly lower than for pure 3F5FPhF6 [27]. MIX5FF6-1, -2, -3 are fragile glassformers, with the fragility index exceeding 50, corresponding to significant deviation of the α-relaxation time from the Arrhenius dependence on temperature. Analysis by the MYEGA model indicated a larger effective activation barrier or smaller number of topological degrees of freedom in MIX5FF6-1, forming glass in the hexatic SmX_A_* phase, compared with MIX5FF6-2, MIX5FF6-3, and 3F5FPhF6 where glass formed in the SmC_A_* phase.
(3) Pure SmX_A_* glass was not obtained in the MHPOBC: 3F5FPhF6 system at moderate cooling rates; MIX5FF6-1 partially crystallized during cooling at 2–40 K/min, and only some fraction of the sample formed the SmX_A_* glass.

In addition to the detailed investigation of the phase sequence in the MHPOBC: 3F5FPhF6 system, the α-relaxation time determined from the dielectric spectra was used to test the application of the VFT, MYEGA, and critical-like models. It was observed that the VFT and MYEGA models produced similar results regarding the glass transition temperature and fragility index. The advantage of the VFT model is its simple formulas for determining these quantities, while for the MYEGA model, the special Lambert W-function is used. On the other hand, the MYEGA model gives better insight into glass transition, as its parameters are attributed to the thermodynamic quantities of the sample. It was also shown that the critical-like model, although suitable for molecules with uniaxial symmetry, applies to chiral molecules, which fulfill this condition only approximately.

## Figures and Tables

**Table 1:** Fitting parameters of the VFT formula for the α-relaxation time in MIX5FF6-1, -2, -3 mixtures. The results for the pure 3F5FPhF6 component from [27] are shown for comparison.

Sample	$\log_{10}\left(\tau_{\infty }/s\right)$	B[K]	$T_{V}$[K]	$T_{g}$[K]	$m_{f}$
MIX5FF6-1	−12.7(3)	1518(95)	187(3)	232.3(4)	76(2)
MIX5FF6-2	−10.4(2)	786(50)	207(2)	234.1(3)	105(4)
MIX5FF6-3	−10.2(3)	743(48)	210(2)	236.1(2)	109(3)
3F5FPhF6 [27]	−11.0(2)	883(50)	208(1)	238.0(3)	105(4)

**Table 2:** Fitting parameters of the MYEGA formula for the α-relaxation time in MIX5FF6-1, -2, -3 mixtures. The results for the pure 3F5FPhF6 component from [27] are shown for comparison.

Sample	$\log_{10}\left(\tau_{\infty }/s\right)$	K[K]	C[K]	$T_{g}$[K]	$m_{f}$
MIX5FF6-1	−10.1(2)	1055(28)	67(10)	231.2(6)	67.1(6)
MIX5FF6-2	−8.5(2)	1549(33)	7(2)	231.4(7)	80.5(7)
MIX5FF6-3	−7.9(2)	1882(28)	1.8(3)	234.7(9)	88.9(4)
3F5FPhF6 [27]	−8.71(8)	1845(25)	2.5(4)	238(2)	94(1)

**Table 3:** Fitting results of the critical-like formula to the α-relaxation time in MIX5FF6-1, -2, -3.

Sample	Trange [K]	$T^{*}$[K]	γ
MIX5FF6-1	249–285	235.4(8)	7.5(2)
MIX5FF6-2	243–289	232(2)	7.5(4)
MIX5FF6-3	243–301	232.3(5)	7.9(1)

## Data Availability

The data associated with this study are available in the RODBUK Cracow Open Research Data Repository, at https://doi.org/10.48733/IFJPAN/SAHUVZ.

## Supplementary Materials

The following supporting information can be downloaded at: https://www.mdpi.com/article/10.3390/ma18133085/s1, Figure S1: POM results of MIX5FF6-1 on cooling; Figure S2: POM results of MIX5FF6-1 on heating; Figure S3: POM results of MIX5FF6-2 on cooling; Figure S4: POM results of MIX5FF6-2 on heating; Figure S5: POM results of MIX5FF6-3 on cooling; Figure S6: POM results of MIX5FF6-3 on heating.

## Abbreviations

The following abbreviations are used in this manuscript:

3F5FPhF6	(S)-4′-(1-methylheptyloxycarbonyl)biphenyl-4-yl 4-[5-(2,2,3,3,4,4,4-heptafluorobutoxy)pentyl-1-oxy]-2,3-difluorobenzoate
BDS	broadband dielectric spectroscopy
DOBAMBC	p-decyloxybenzylidene p’-amino 2-methyl butyl cinnamate
DSC	differential scanning calorimetry
GM	Goldstone mode
MHPOBC	(S)-4-[(1-methylheptyloxy)carbonyl]phenyl 4′-octyloxy-4-biphenylcarboxylate
MIX5FF6-1	MHPOBC: 3F5FPhF6 mixture with 0.9: 0.1 molar ratio
MIX5FF6-2	MHPOBC: 3F5FPhF6 mixture with 0.75: 0.25 molar ratio
MIX5FF6-3	MHPOBC: 3F5FPhF6 mixture with 0.5: 0.5 molar ratio
MWS	Maxwell–Wagner–Sillars
MYEGA	Mauro–Yue–Ellison–Gupta–Allan
ODIC	orientationally disordered crystal
POM	polarizing optical microscopy
SM	soft mode
VFT	Vogel–Fulcher–Tammann
XRD	X-ray diffraction
