# Supplementary material for: Partially Disordered Crystal Phases and Glassy Smectic Phases in Liquid Crystal Mixtures
Source: Materials (Basel). 2025 Jun 29;18(13):3085. doi: 10.3390/ma18133085 (PMC12251179; doi:10.3390/ma18133085)
Supplement: Supplementary file 1 [file materials-18-03085-s001.zip › materials-3711115-supplementary.pdf]

Partially disordered crystal phases and glassy smectic phases in liquid crystal mixtures

Aleksandra Deptuch[1,*], Anna Drzewicz[1], Magdalena Urbańska[2], Ewa Juszyńska-Gałązka[1,3]

[1] Institute of Nuclear Physics Polish Academy of Sciences, Radzikowskiego 152, PL-31342 Kraków, Poland

[2] Institute of Chemistry, Military University of Technology, Kaliskiego 2, PL-00908 Warsaw, Poland

[3] Research Center for Thermal and Entropic Science, Graduate School of Science, Osaka University, 560-0043 Osaka, Japan

*corresponding author, aleksandra.deptuch@ifj.edu.pl

Supplementary Materials

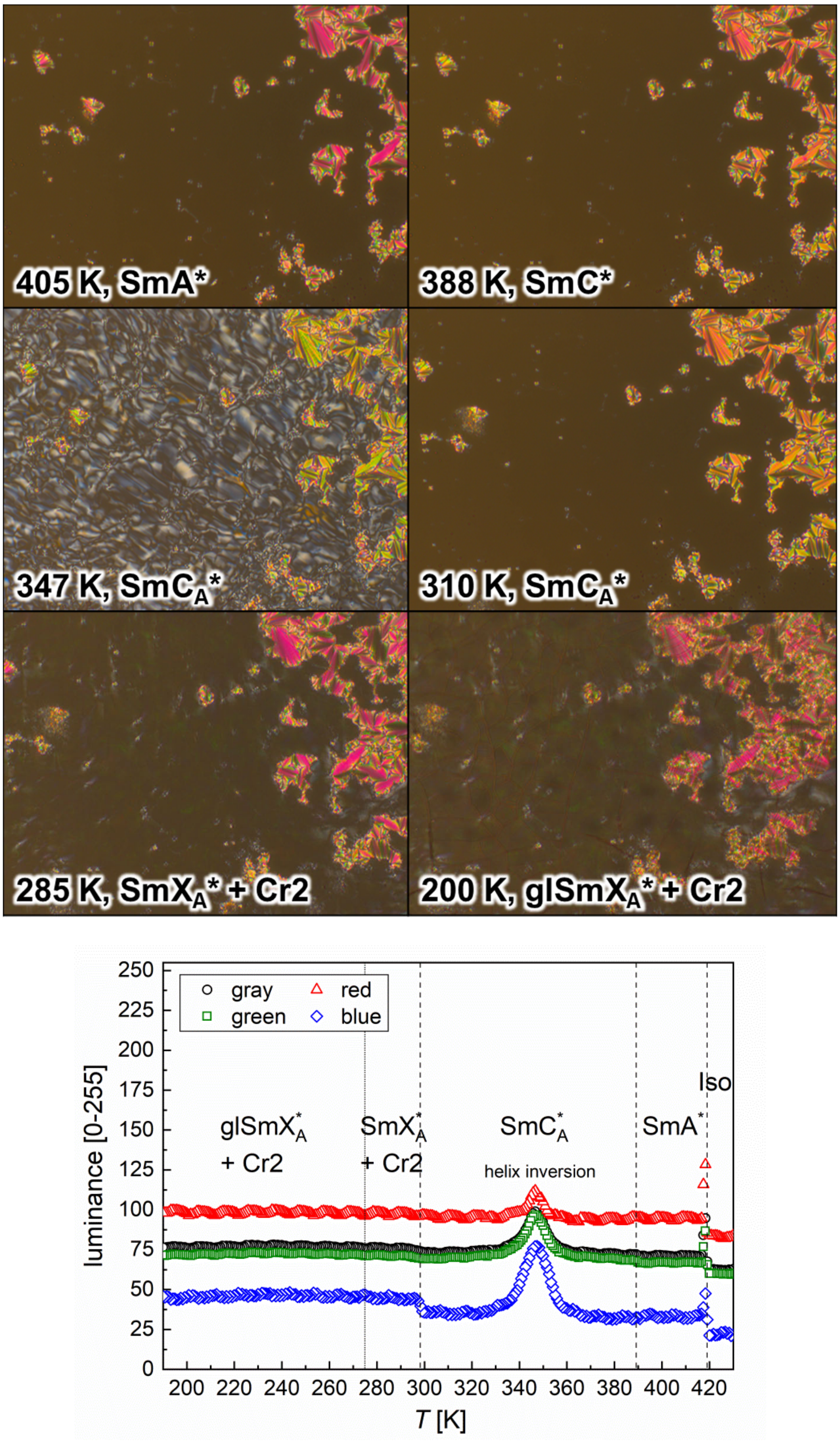

Figure S1. Representative POM textures (622 × 466 μm^2) of MIX5FF6-1 collected at the 10 K/min cooling rate as well as the red, green, blue components and weighted total luminance of each texture. The glass transition temperature is based on DSC thermograms at the same rate.

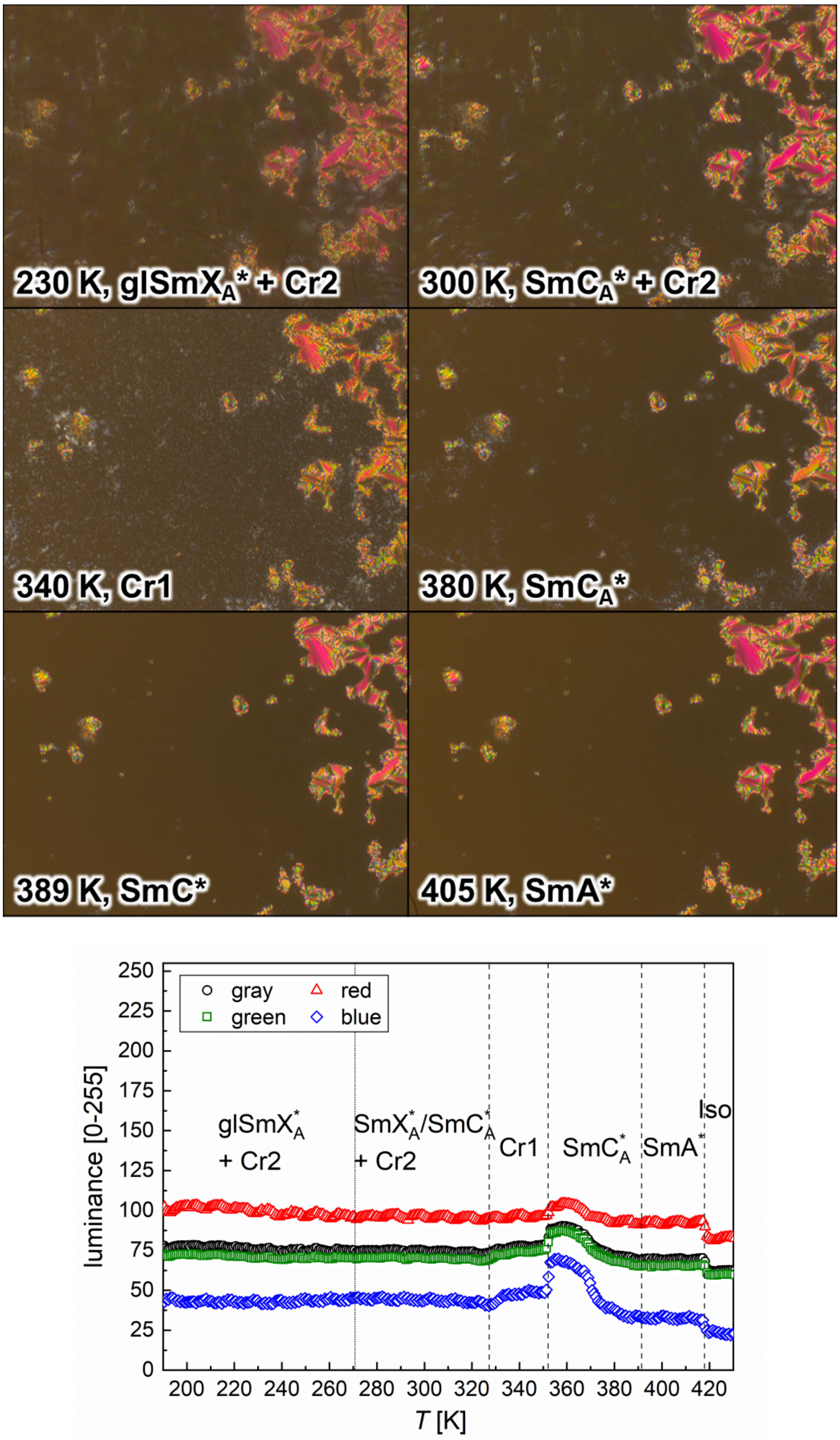

Figure S2. Representative POM textures (622 × 466 μm^2) of MIX5FF6-1 collected at the 10 K/min cooling rate as well as the red, green, blue components and weighted total luminance of each texture. The glass transition temperature is based on DSC thermograms at the same rate.

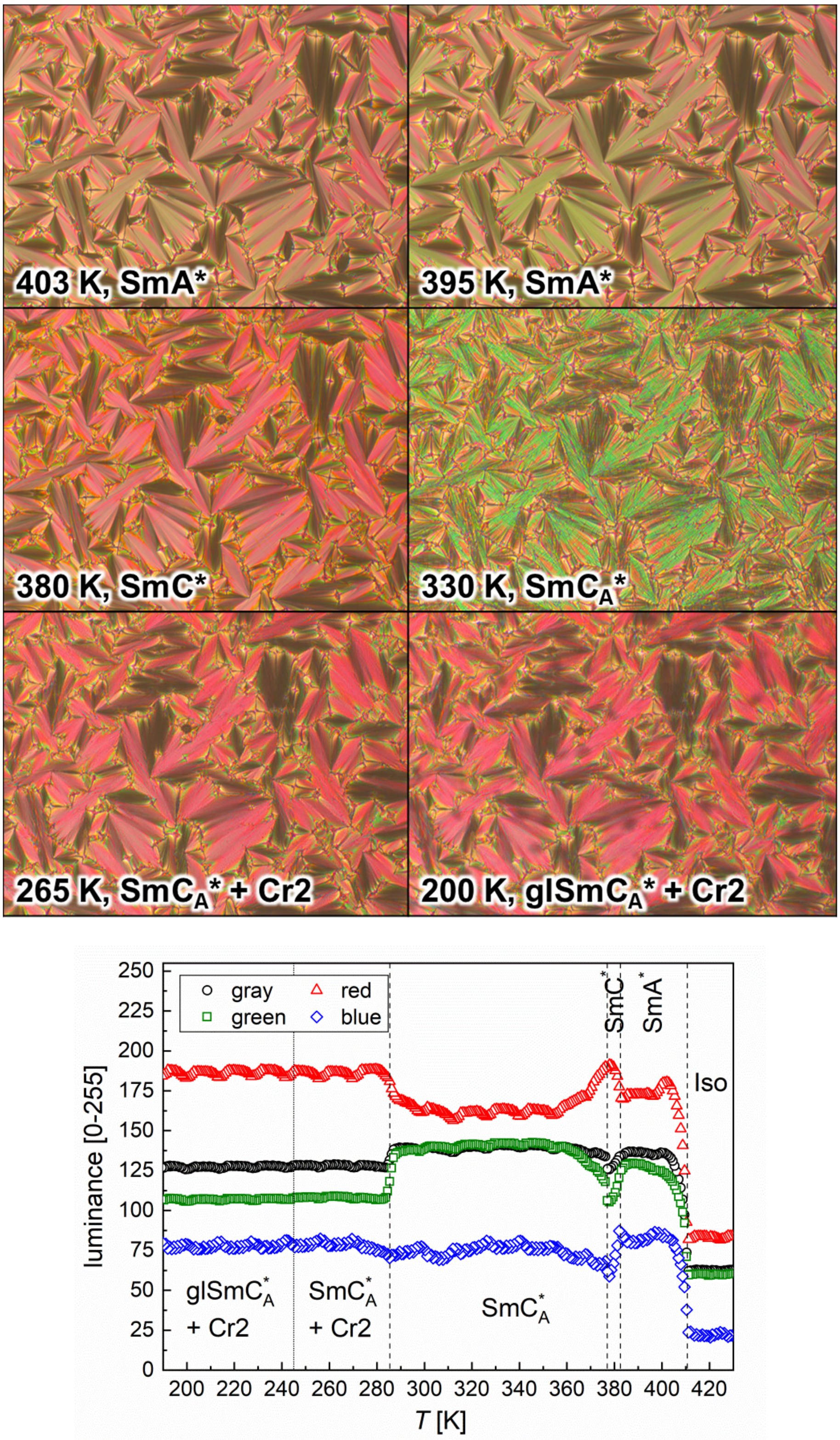

Figure S3. Representative POM textures (622 × 466 μm^2) of MIX5FF6-2 collected at the 10 K/min cooling rate as well as the red, green, blue components and weighted total luminance of each texture. The glass transition temperature is based on DSC thermograms at the same rate.

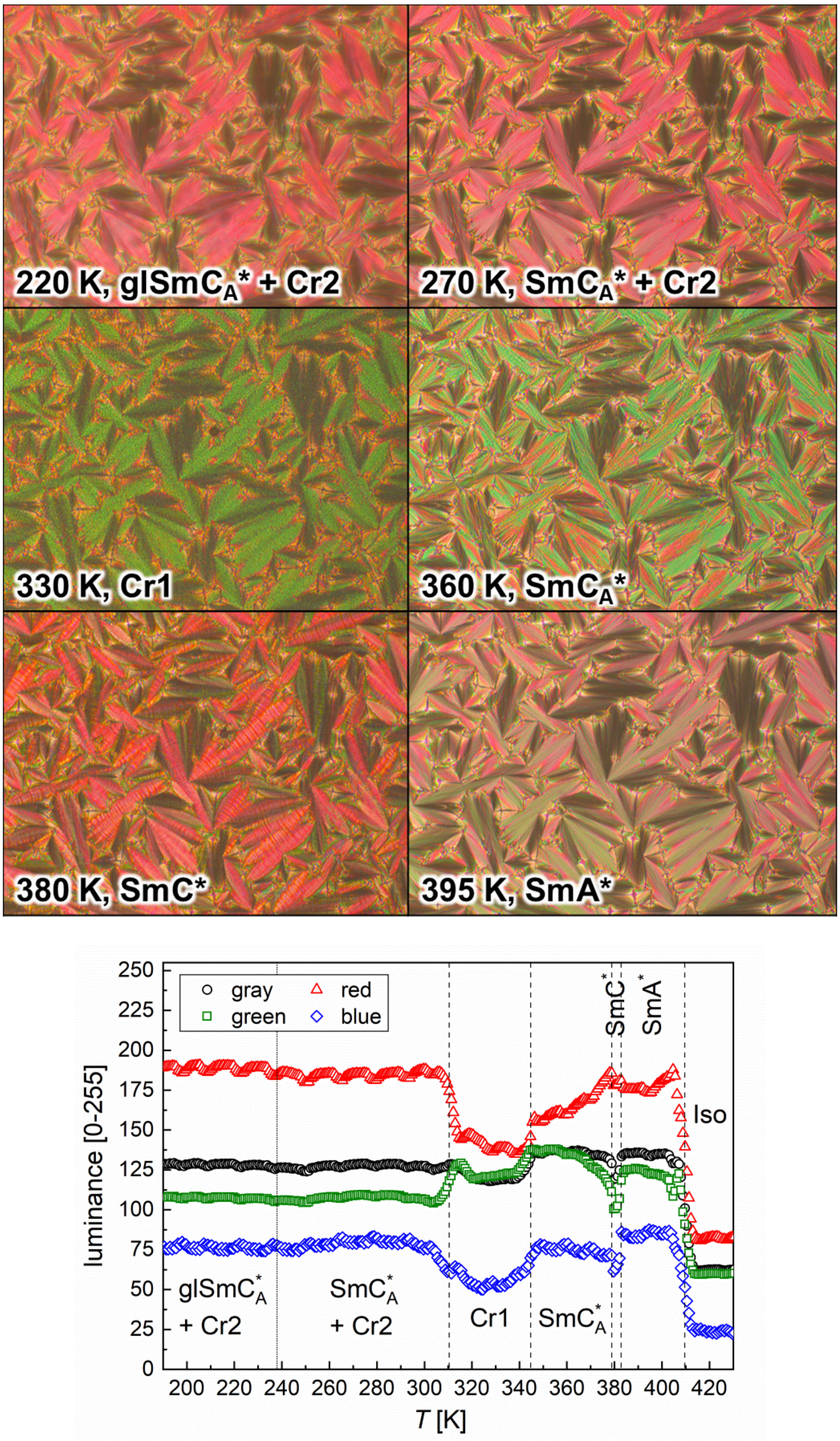

Figure S4. Representative POM textures (622 × 466 μm^2) of MIX5FF6-2 collected at the 10 K/min heating rate as well as the red, green, blue components and weighted total luminance of each texture. The glass transition temperature is based on DSC thermograms at the same rate.

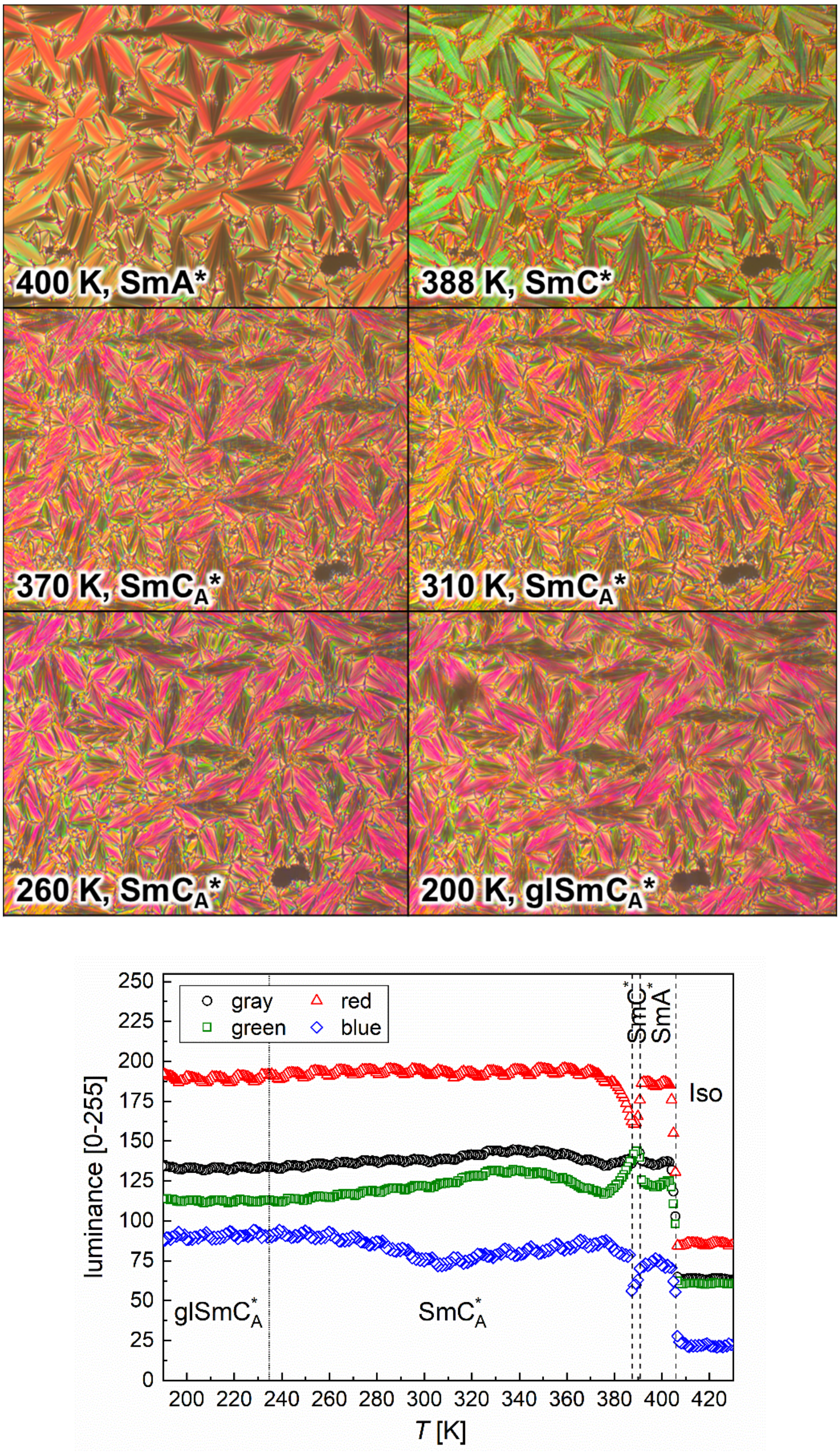

Figure S5. Representative POM textures (622 × 466 μm^2) of MIX5FF6-3 collected at the 10 K/min cooling rate as well as the red, green, blue components and weighted total luminance of each texture. The glass transition temperature is based on DSC thermograms at the same rate.

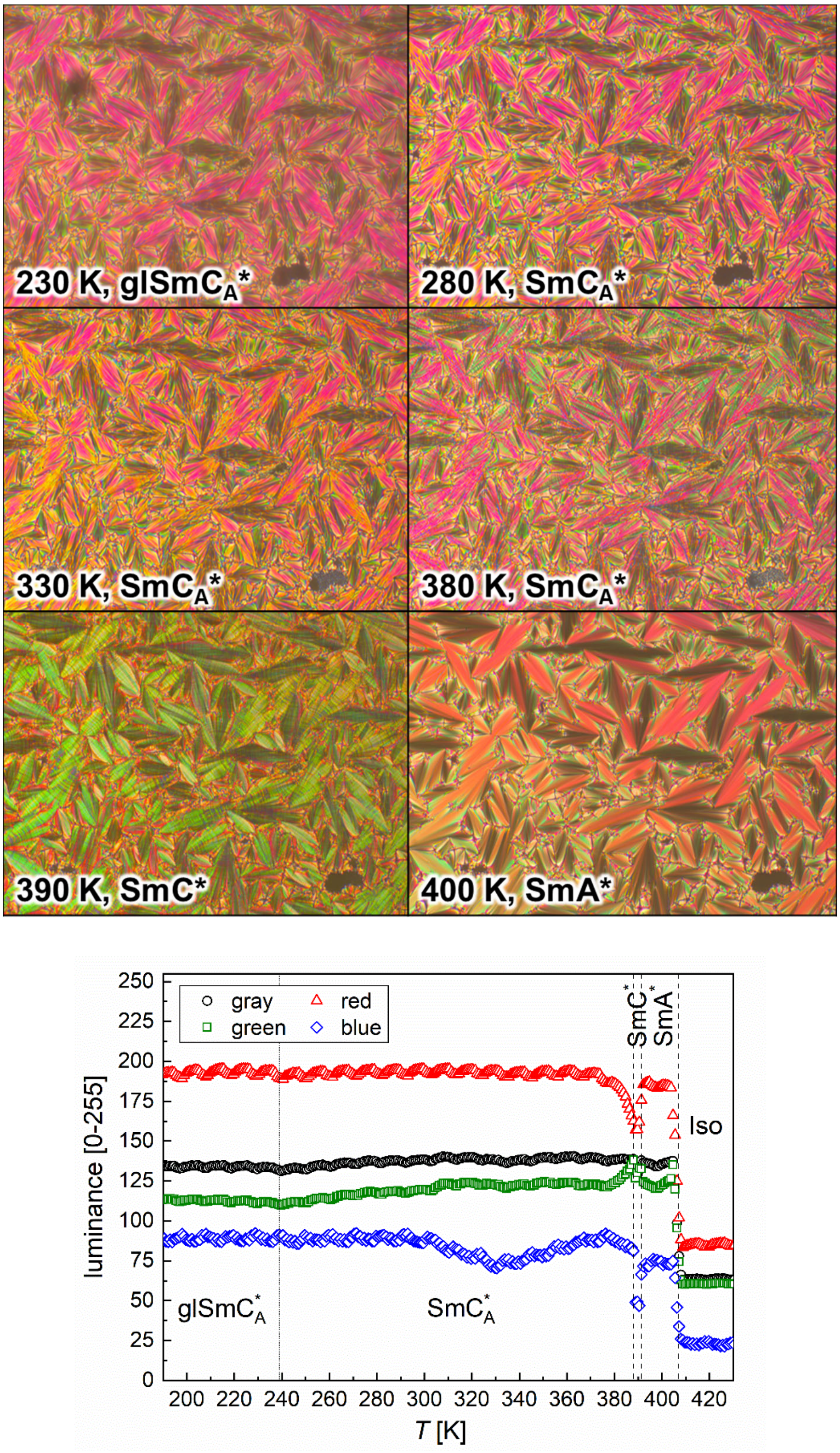

Figure S6. Representative POM textures (622 × 466 μm^2) of MIX5FF6-3 collected at the 10 K/min heating rate as well as the red, green, blue components and weighted total luminance of each texture. The glass transition temperature is based on DSC thermograms at the same rate.
